# A Reconstruction Approach for Imaging in 3D Cone Beam Vector Field Tomography

**DOI:** 10.1155/2008/174283

**Published:** 2009-02-03

**Authors:** T. Schuster, D. Theis, A. K. Louis

**Affiliations:** ^1^Department of Mechanical Engineering, Helmut Schmidt University, Holstenhofweg 85, 22043 Hamburg, Germany; ^2^Department of Applied Mathematics, Saarland University, P.O. Box 15 11 50, 66041 Saarbrücken, Germany

## Abstract

3D cone beam vector field tomography (VFT) aims for reconstructing and visualizing the velocity field of a moving fluid by measuring line integrals of projections of the vector field. The data are obtained by ultrasound measurements along a scanning curve which surrounds the object. From a mathematical point of view, we have to deal with the inversion of the vectorial cone beam transform. Since the vectorial cone beam transform of any gradient vector field with compact support is identically equal to zero, we can only hope to reconstruct the solenoidal part of an arbitrary vector field. In this paper we will at first summarize important properties of the cone beam transform for three-dimensional solenoidal vector fields and then propose a solution approach based on the method of approximate inverse. In this context, we intensively make use of results from scalar 3D computerized tomography. The findings presented in the paper will continuously be illustrated by pictures from first numerical experiments done with exact, simulated data.

## 1. INTRODUCTION

Vector field tomography (VFT) deals with the problem of reconstructing a vector field, for example, a velocity field of an incompressible, moving fluid, from line integrals of projections of the field. VFT has various applications in photoelasticity, oceanography, nondestructive testing, and medical imaging, where we may think of tumor detection by reconstructing and visualizing blood flow which is known to be more irregular and more intense around tumors than in normal tissue, see [1]. The integral data can be measured using ultrasound signals when we assume that the Doppler shift of the frequency is approximately proportional to the velocity of the particle in the fluid which causes the shift. This is a reasonable assumption if the particle velocity is significantly smaller than the speed of sound within the medium under consideration.

Although this seems to be quite simple at first sight it will become clear after the definition of the cone beam transform for vector fields that only the projection of the vector field onto the line of integration can be measured. This enormous loss of information is the reason why we can only hope to recover the solenoidal part of the vector field from our measurements, a fact which has for example been shown in [2].

Ultrasound devices may be utilized as a supportive method in preliminary examinations for tumor detection by reconstructing and visualizing blood flow which has already been suggested in 1977 by Wells et al. [3]. This may help to reduce the radiation dose of a patient tremendously. Thinking of mammography, it is known that the pressure which is put on the breast during the examination may crush small existing tumors and allow them to spread more easily. This danger could also be avoided or at least reduced by using ultrasound for preventive medical examinations. It should be noted that these deliberations are subject to the condition that the algorithms and the medical equipment based on ultrasound work as reliable and fast as current X-ray techniques.

**Figure 1:**
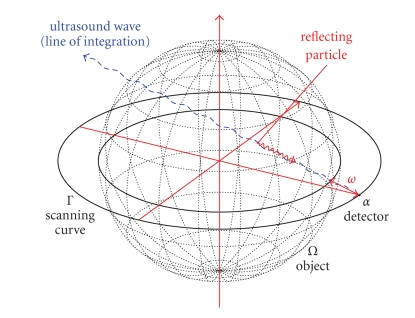
Measurement setup using a circle in the plane {*x*_3_ = 0} as scanning curve Γ.

A possible measurement setup where the scanning curve Γ is a circle in the plane {*x*_3_ = 0} is depicted in Figure 1. This defines exactly the geometry used in our first numerical experiments which are presented in the last section of this work. A lot of theoretical and numerical results have been achieved over the last few years for the parallel geometry. Juhlin [4] suggested a measurement setup which is suited to fully reconstruct solenoidal fields in two dimensions. Mathematical properties of this model can be found in Sparr et al. [5]. The singular value decomposition for the 2D fan-beam Radon transform of tensor fields has been presented in an article by Kazantsev and Bukhgeim [6]. Desbat and Wernsdörfer [7] developed an iterative method. For 3D Doppler tomography, Schuster established an inversion scheme of filtered backprojection type [8, 9] relying on the method of approximate inverse. Together with Rieder [10], he obtained convergence with rates and stability with respect to noisy data for this method.

As in scalar 3D computerized tomography, the *cone beam transform* is of special interest from a practical point of view. It is defined for a tensor field of rank *m* by

(1) $$\begin{array}{ll} D_{m}f(\alpha ,\omega ) & =\int_{0}^{\infty }\langle f(\alpha +t\omega ),\omega^{m}\rangle \text{d}t\\ & =\int_{0}^{\infty }f_{i_{1}\cdots i_{m}}(\alpha +t\omega )\omega^{i_{1}}\cdots \omega^{i_{m}}\text{d}t, \end{array}$$

where *α* ∈ Γ is a source point on the scanning curve $\Gamma \subset {\mathbb{R}}^{n}\setminus \overset{¯}{\Omega }$ which surrounds the object Ω, *ω* ∈ *S* ^*n*−1^ is the unit vector of direction of the line and **f** is a tensor field of rank *m* with compact support in the open domain Ω. Tensor fields of rank *m* = 0 are scalar functions *f*(*x*), tensor fields of rank *m* = 1 are vector fields **f**(*x*) in ℝ^*n*^. In (1) we use Einstein's summation rule, that means we sum up over equal indices *i*_*j*_, where 1 ≤ *i*_*j*_ ≤ *n*.

A lot of theory has been developed for the common case of tensor fields of arbitrary rank. To facilitate notation and to direct the readers' attention to the cases important for practical applications we confine the following remarks to the *scalar cone beam transform* as well as the *cone beam transform for vector fields* in *n* dimensions. In the following, scalar fields will be denoted by *f* whereas vector fields will be written bold-faced, such as **f**.

Setting *m* = 0 in (1) we obtain the well-known *(scalar) cone beam transform*

(2) $$D_{0}f(\alpha ,\omega )=\int_{0}^{\infty }f(\alpha +t\omega )\text{d}t$$

of a scalar field *f* : ℝ^*n*^ ⊃ Ω → ℝ. For *m* = 1 formula (1) is the *cone beam transform for vector fields* **f** : ℝ^*n*^ ⊃ Ω → ℝ^*n*^, which in 3D is also often referred to as the *Doppler transform*. It reads as

(3) $$D_{1}f(\alpha ,\omega )=\int_{0}^{\infty }\langle f(\alpha +t\omega ),\omega \rangle \text{d}t.$$

Hence the mathematical problem of 3D cone beam VFT consists of inverting **D**_1_ **f** = *g* for given measurements *g* ∈ ℝ, that is reconstructing a three-dimensional vector field **f** from one-dimensional, that is, scalar, data *g*. In contrast to **D**_0_ an inversion formula for **D**_1_ is not known by now and an inversion scheme for the cone beam transform for vector fields has not been established so far.

**Figure 2:**
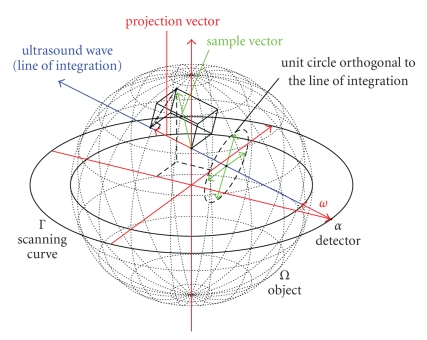
Visualization of the measured projection data for **D**_1_ **f**.

From (3) it can be seen that only the integral of the projections of the vector field along the ray of integration can be obtained from the measurements, which is emphasized in Figure 2. Considering an ultrasound wave starting from the source point *α* on the scanning curve Γ only the projection of the green sample vector onto the line of integration can be measured. The projection is illustrated by the red arrow. This also means that any vector orthogonal to the ultrasound wave does not contribute to the integral at all, a property which is depicted by the unit circle and the corresponding sample vectors orthogonal to *ω*. As a consequence, a full reconstruction of an arbitrary vector field is impossible with the underlying measurement geometry. As already mentioned in the abstract of our paper, we can only hope to reconstruct the solenoidal part of an arbitrary vector field since the vectorial cone beam transform of any gradient vector field with compact support is identically equal to zero. This can easily be seen from the following short computation for a gradient field **f**(*x*) = ∇*ϕ*(*x*) = (∂_*x*_1__ *ϕ*(*x*),…, ∂_*x*_*n*__ *ϕ*(*x*))^⊤^, *ϕ* : ℝ^*n*^ ⊃ Ω → ℝ, with compact support

(4) $$\begin{array}{ll} D_{1}f(\alpha ,\omega ) & =D_{1}\left(\nabla \phi \right)(\alpha ,\omega )=\int_{0}^{\infty }\nabla \phi \left(\alpha +t\omega \right)\omega \;\text{d}t\\ & ={\left[\phi (\alpha +t\omega )\right]}_{0}^{\infty }=0. \end{array}$$

The method of *approximate inverse* introduced by Louis and Maass [11] delivers a mathematical framework for coping with inverse problems in an efficient way. The method computes a smoothed version of the solution **f** with the help of so-called *mollifiers*. These are smooth approximations to delta functions. Using a duality argument the method then consists of evaluations of inner products of the given data *g* with *reconstruction kernels*. One feature of the method is, that invariances of the underlying operator can be used to speed up the computation time tremendously. This method was successfully applied to the reconstruction problem in 3D computerized tomography, that is, (2), see [12]. We use these results to extend the method of approximate inverse to (3).

We summarize the contents of the paper. First we state essential mathematical properties of (1) for the special cases of *m* = 0 and *m* = 1. The interested reader will find generalizations of the presented theorems to symmetric, covariant tensor fields of any rank and in any dimension in [13], especially the extension of Grangeat's formula which has been proven for (1) in case *n* = 3, *m* = 0 (see (2)) in [14]. Then we outline how the method of approximate inverse can be used for (2) to solve **D**_0_ *f* = *g* and present its application to (3) to solve **D**_1_ **f** = *g*. Furthermore, an approach is presented, how reconstruction kernels for **D**_1_ from (3) can be calculated with the help of known reconstruction kernels for the scalar cone beam transform **D**_0_. Some pictures from numerical experiments show that this approach is very promising.

## 2. MATHEMATICAL PROPERTIES OF **D**_0_ AND **D**_1_

Let

(5) $$L^{2}(X,{\mathbb{R}}^{n}):=\{f:X\to {\mathbb{R}}^{n}:∥f{∥}_{L^{2}}={\langle f,f\rangle }_{L^{2}}^{1/2}<\infty \}$$

denote the space of square integrable functions from *X* ⊂ ℝ^*n*^ to ℝ^*n*^ where the *L* ^2^-inner product of two functions is given as

(6) $${\langle f,g\rangle }_{L^{2}}=\int_{X}{\langle f(x),g(x)\rangle }_{{\mathbb{R}}^{n}}\text{d}x.$$

Fundamental properties of (2) and (3) are summarized in the following theorem, which is the result of straightforward calculations.

**Theorem 1**

*Let* Ω^*n*^ := {*x* ∈ ℝ^*n*^ : |*x*| < 1} *with* ∂Ω^*n*^ = *S* ^*n*−1^ *the reconstruction region, that is, the region in which the object* Ω *is contained. The mappings*

(7) $$\begin{array}{ll} D_{0} & :L^{2}(\Omega^{n})\to L^{2}(\Gamma \times S^{n-1}),\\ D_{1} & :L^{2}(\Omega^{n},{\mathbb{R}}^{n})\to L^{2}(\Gamma \times S^{n-1}) \end{array}$$

*are linear and bounded if*

(8) $$\int_{\Gamma }{\left(|\alpha |-1\right)}^{1-n}\text{d}\alpha <\infty .$$

*The adjoints (*backprojections*)* **D**_0_^∗^ : *L* ^2^(Γ × *S* ^*n*−1^) → *L* ^2^(Ω^*n*^) *and* **D**_1_^∗^ : *L* ^2^(Γ × *S* ^*n*−1^) → *L* ^2^(Ω^*n*^, ℝ^*n*^) *are given by*

(9) $$\begin{array}{ll} D_{0}^{*}g(x) & =\int_{\Gamma }\{|x-\alpha {|}^{1-n}g(\alpha ,\frac{x-\alpha }{\left|x-\alpha \right|})\}\text{d}\alpha ,\\ D_{1}^{*}g(x) & =\int_{\Gamma }\{|x-\alpha {|}^{-n}g(\alpha ,\frac{x-\alpha }{\left|x-\alpha \right|})(x-\alpha )\}\text{d}\alpha . \end{array}$$

For *n* = 3, we obtain the well-known cone beam transform **D**_0_ with the corresponding backprojection operator **D**_0_^∗^ of scalar fields which is thoroughly investigated in 3D computerized tomography as well as the mathematical model of 3D cone beam vector tomography and the backprojection reads as

(10) $$D_{1}^{*}g(x)=\int_{\Gamma }|x-\alpha {|}^{-2}g(\alpha ,\frac{x-\alpha }{\left|x-\alpha \right|})\frac{x-\alpha }{\left|x-\alpha \right|}\text{d}\alpha .$$

**D**_1_^∗^ *g*(*x*) represents an integration over all lines intersecting *x*. The result can be seen as an average over all directions connecting a source point *α* and *x*.

One of the crucial tools when computing reconstruction kernels in scalar cone beam tomography is the formula of Grangeat [14]. We proved a generalization of that formula which is valid for any symmetric, covariant tensor field of rank *m* in *n* dimensions in [13] but our presentation will be restricted to **D**_0_ and **D**_1_.

**Theorem 2 (Schuster [13] based on Hamaker et al. [15]).**

*Assume* *n* ≥ 2, *f* ∈ *𝒞*_0_^(*n*−2)^(Ω^*n*^), *and* **f** ∈ *𝒞*_0_^(*n*−2)^(Ω^*n*^, ℝ^*n*^). *Then,*

(11) $$\begin{array}{ll} & \frac{\partial^{\left(n-2\right)}}{\partial s^{\left(n-2\right)}}Rf(\omega ,\langle \alpha ,\omega \rangle )\\ & \;\;=(-{1)}^{\left(n-2\right)}\int_{S^{n-1}}D_{0}f(\alpha ,\theta )\delta^{\left(n-2\right)}(\langle \omega ,\theta \rangle )\text{d}S(\theta ), \end{array}$$

(12) $$\begin{array}{ll} & \frac{\partial^{\left(n-2\right)}}{\partial s^{\left(n-2\right)}}Rf_{\alpha }(\omega ,\langle \alpha ,\omega \rangle )\\ & \;\;=(-{1)}^{\left(n-2\right)}\int_{S^{n-1}}D_{1}f(\alpha ,\theta )\delta^{\left(n-2\right)}(\langle \omega ,\theta \rangle )\text{d}S(\theta ), \end{array}$$

*where* *α* ∈ Γ, *ω* ∈ *S* ^*n*−1^, d*S* *denotes the surface measure on* *S* ^*n*−1^, **R** *is the *n*-dimensional *Radon transform* defined by*

(13) $$(Rf)(\theta ,s)=\int_{\langle x,\theta \rangle =s}f(x)\text{d}x,$$

*and*

(14) $$f_{\alpha }(x)={\langle f(x),|x-\alpha {|}^{-1}(x-\alpha )\rangle }_{{\mathbb{R}}^{n}}$$

*is the projection of* **f** *onto* (*x* − *α*)/ |*x* − *α*|.

In the following, we want to limit our presentation to *n* = 3. Then formula (12) reads as

(15) $$\begin{array}{ll} \frac{\partial }{\partial s}Rf_{\alpha }(\omega ,\langle \alpha ,\omega \rangle ) & =(-1)\int_{S^{2}}D_{1}f(\alpha ,\theta )\delta^{'}(\langle \omega ,\theta \rangle )\text{d}S(\theta )\\ & =\int_{S^{2}\cap \{\langle \theta ,\omega \rangle =0\}}\langle \nabla_{y}D_{1}f(\alpha ,y=\theta ),\omega \rangle \text{d}S(\theta ). \end{array}$$

In the scalar case a solver for **D**_0_ can be constructed with the help of (11) for *n* = 3. This is done by inverting the Radon transform **R** which is possible if the condition of Tuy-Kirillov is satisfied. It tells that we have full knowledge of **R** *g*(*ω*, *s*) for all *ω*, *s* and any scalar function *g* : Ω^3^ → ℝ, if any plane intersecting the object Ω ⊆ Ω^3^ does also have at least one intersection point with the scanning curve Γ and this intersection must be nontransversally. This works fine for **D**_0_ since then *f*(*x*) is independent of *α* but unfortunately that does not help in case of **D**_1_ (and analogous transforms for tensor fields as well, see [13]), since there the object function *f*_*α*_ = 〈**f**(*x*), (*x* − *α*)/|*x* − *α*|〉_ℝ^3^_ of **R** depends on and hence changes with *α*, see (15). Thus we seek an alternative way of solving **D**_1_ **f** = *g* for vector fields **f**.

## 3. APPROXIMATION OF RECONSTRUCTION KERNELS IN VECTOR FIELD TOMOGRAPHY

As already said, an inversion formula for **D**_1_ is not known by now and an inversion scheme for the cone beam transform for vector fields has not been established so far. So, the aim of this paper is to deduce a completely new method for three-dimensional cone beam VFT. A comparison with existing algorithms is difficult since there are no inversion methods for the vectorial cone beam transform to the authors' best knowledge. Nevertheless, some famous algorithms will certainly come to the reader's mind when thinking of tomography. The well-known FDK algorithm (Feldkamp et al. see [16]) is detailed on [17, page 128]. From this description it immediately becomes clear that the algorithm does not work for **D**_1_. The integrand of the cone beam transform for vector fields strongly depends on the direction *ω*, a fact which is explicitly disregarded by the FDK algorithm. The methods of Norton (see [18, 19]) and Prince [20] are specifically suited to solve 2D, respectively, 3D problems for vector fields in parallel geometry. They both use transforms different from **D**_1_. The generalization of Norton's approach to 3D vector tomography of Doppler-transformed fields in parallel geometry was a challenging problem for Lade et al. in [21]. Regrettably, neither approach can be adapted to VFT using the cone beam geometry. Finally, no Fourier slice theorem for VFT is known, not even for standard 3D cone beam tomography, so Fourier methods are not an alternative.

The method of *approximate inverse*, which was established by Louis and Maass [11] in 1990, leads to an algorithm of filtered backprojection type if invariances and some appropriate approximations are used. This has been shown for example in [22, 23] or [12]. Fundamental properties of it have also been published in [24, 25]. Its theory was enhanced over the last decade and the method was successfully applied to several reconstruction problems in medical imaging and nondestructive testing, such as computerized tomography, inverse scattering, thermoacoustic computerized tomography, diffractometry, and Doppler tomography. In [12] the method was applied to 3D cone beam tomography, that is, to **D**_0_. We describe this approach and then formulate an extension of it to **D**_1_.

Let *f* ∈ *L* ^2^(Ω^3^) be a scalar function. The approximate inverse computes a smoothed version *f*_*γ*_ of *f* by convolving *f* with a *mollifier* *e*_*γ*_ ∈ *𝒞* ^∞^(ℝ^3^). A mollifier *e*_*γ*_ is a smooth function with small essential support having the property that

(16) $$f_{\gamma }(x):=(f*e_{\gamma })(x)\to f(x)\;\text{as}\;\;\gamma \to 0.$$

Here, ∗ denotes the convolution

(17) $$(f*h)(x)=\int_{{\mathbb{R}}^{3}}f(y-x)h(y)\text{d}y.$$

Such a function is given by the Gaussian kernel

(18) $$e_{\gamma }(x)=\frac{\gamma^{-3}}{{\left(2\pi \right)}^{3/2}}\exp\left(-\frac{|x{|}^{2}}{2\gamma^{2}}\right).$$

Provided that we can solve the equation

(19) $$D_{0}^{*}[v_{\gamma }(x)]=e_{\gamma }(x-\cdot ),$$

then we can reconstruct *f*_*γ*_ from the measured cone beam data **D**_0_ *f* by

(20) $$\begin{array}{ll} f_{\gamma }(x) & ={\langle D_{0}f,v_{\gamma }(x)\rangle }_{L^{2}(\Gamma \times S^{2})}\\ & =\int_{\Gamma }\int_{S^{2}}(D_{0}f)(\alpha ,\omega )v_{\gamma }(x;\alpha ,\omega )\text{d}S(\omega )\text{d}\alpha , \end{array}$$

where *v*_*γ*_(*x*) = *v*_*γ*_(*x*; *α*, *ω*) ∈ *L* ^2^(Γ × *S* ^2^) for *x* ∈ Ω^*n*^ is called a *reconstruction kernel*. Hence the method of approximate inverse consists of evaluating inner products of the given data **D**_0_ *f* with reconstruction kernels *v*_*γ*_(*x*). This can be done in an efficient way using the translation invariance of *e*_*γ*_ and some appropriate approximations which are outlined in detail in [12]. These imply that we have to solve (19) only once, namely for *x* = 0, and apply the invariances to get the remaining reconstruction kernels. By doing so the computation time is shortened significantly. The computation of reconstruction kernels for circular 3D cone beam tomography, that is, for exactly the same scanning geometry that we use for the reconstruction of vector fields, has been detailed in [26].

In comparison to other regularization methods such as Tikhonov regularization which would result in enormous computational costs because of the very large, full matrices, the approximate inverse is much more efficient, an advantage that is especially crucial in tomographic applications because of the large number of evaluations.

To apply the method to **D**_1_ and hence to VFT, we construct mollifier fields **E**_*γ*_^*j*^ ∈ *L* ^2^(Ω^3^, ℝ^3^) defining

(21) $$E_{\gamma }^{j}(x):=e_{\gamma }(x)\cdot e_{j},\;j\in \{1,2,3\},$$

where *e*_1_ = (1, 0, 0)^⊤^, *e*_2_ = (0, 1, 0)^⊤^ and *e*_3_ = (0, 0, 1)^⊤^. Using again the Gaussian (18) as mollifier *e*_*γ*_ we obtain

(22) $${\left(f_{\gamma }\right)}_{j}(x):=(f*E_{\gamma }^{j})(x)\to f_{j}(x)\;\text{as}\;\;\gamma \to 0$$

for **f** ∈ *L* ^2^(Ω^3^, ℝ^3^). Unfortunately, by now the exact reconstruction kernels **V**_*γ*_^*j*^(*x*), that is, the solutions of **D**_1_^∗^[**V**_*γ*_^*j*^(*x*)] = **E**_*γ*_^*j*^(*x* − ·) are still unknown. But the special structure of the mollifier fields **E**_*γ*_^*j*^ allow for a computation of reconstruction kernels for

(23) $$Pf(\alpha ,\omega )=\int_{0}^{\infty }f(\alpha +t\omega )\text{d}t$$

with the help of kernels for **D**_0_.

**Theorem 3**

*Let* *v*_*γ*_ *be the reconstruction kernel associated to* *e*_*γ*_ *with respect to* **D**_0_, *that is,*

(24) $$D_{0}^{*}[v_{\gamma }(x)]=e_{\gamma }(x-\cdot ).$$

*Defining* **V**_*γ*_^*j*^(*x*; *α*, *ω*) := *v*_*γ*_(*x*; *α*, *ω*)·*e*_*j*_ ∈ *L* ^2^(Γ × *S* ^2^, ℝ^3^) *yields*

(25) $$P^{*}[V_{\gamma }^{j}(x)]=E_{\gamma }^{j}(x-\cdot ),$$

*that means* **V**_*γ*_^*j*^ *is a reconstruction kernel associated to* **E**_*γ*_^*j*^ *with respect to* **P**. *The adjoint* **P** ^∗^ *of* **P** *is given as*

(26) $$P^{*}g(x)=\int_{\Gamma }|x-\alpha {|}^{-2}g(\alpha ,\frac{x-\alpha }{\left|x-\alpha \right|})\text{d}\alpha$$

*for* **g** ∈ *L* ^2^(Γ × *S* ^2^, ℝ^3^).

**Proof**

The adjoint **P** ^∗^ is computed as

(27) $$\begin{array}{ll} {\langle Pf,g\rangle }_{L^{2}(\Gamma \times S^{2},{\mathbb{R}}^{3})} & =\int_{\Gamma }\int_{S^{2}}\int_{0}^{\infty }\langle f(\alpha +t\omega ),g(\alpha ,\omega )\rangle \text{d}t\;\text{d}S(\omega )\text{d}\alpha \\ & =\int_{\Gamma }\int_{R^{3}}\langle f(x),g(\alpha ,\frac{x-\alpha }{\left|x-\alpha \right|})\rangle |x-\alpha {|}^{-2}\text{d}x\;\text{d}\alpha \\ & ={\langle f,P^{*}g\rangle }_{L^{2}(\Omega ,{\mathbb{R}}^{3})}, \end{array}$$

where we applied Fubini's theorem as well as the substitution *x* = *α* + *t* *ω*. A short calculation further shows that

(28) $$\begin{array}{ll} P^{*}[V_{\gamma }^{j}(x)](y) & =\int_{\Gamma }|y-\alpha {|}^{-2}V_{\gamma }^{j}(x;\alpha ,\frac{y-\alpha }{\left|y-\alpha \right|})\text{d}\alpha \\ & =\int_{\Gamma }|y-\alpha {|}^{-2}v_{\gamma }(x;\alpha ,\frac{y-\alpha }{\left|y-\alpha \right|})\text{d}\alpha \cdot e_{j}\\ & =D_{0}^{*}[v_{\gamma }(x)](y)\cdot e_{j}=e_{\gamma }(x-y)\cdot e_{j}\\ & =E_{\gamma }^{j}(x-y). \end{array}$$

The data **Pf** are not known and cannot be computed from **D**_1_ **f**. But, observing that

(29) $$D_{1}f(\alpha ,\omega )={\langle Pf(\alpha ,\omega ),\omega \rangle }_{{\mathbb{R}}^{3}}$$

and since **Pf**(*α*, *ω*) ∈ ℝ^3^ we obtain

(30) $$Pf(\alpha ,\omega )=D_{1}f(\alpha ,\omega )\omega +\lambda_{1}(\alpha ,\omega_{1}^{⊥})\omega_{1}^{⊥}+\lambda_{2}(\alpha ,\omega_{2}^{⊥})\omega_{2}^{⊥},$$

where *ω*_1_^⊥^, *ω*_2_^⊥^ ∈ *S* ^2^ are such that {*ω*, *ω*_1_^⊥^, *ω*_2_^⊥^} is an orthonormal basis of ℝ^3^ and *λ*_1_, *λ*_2_ are appropriate coefficients. Thus approximating

(31) $$Pf(\alpha ,\omega )\approx D_{1}f(\alpha ,\omega )\omega$$

we neglect the parts orthogonal to *ω* and can apply the method of approximate inverse using the reconstruction kernels **V**_*γ*_^*j*^ for **P**. This procedure results in Algorithm 1.

It is worth to mention that the mathematical model has not been changed. Results for the scalar cone beam transform **D**_0_ *f*(*α*, *ω*) are transferred to its 3D equivalent **Pf**(*α*, *ω*) which is an approximation to **D**_1_ **f**(*α*, *ω*) by using **Pf**(*α*, *ω*) ≈ **D**_1_ **f**(*α*, *ω*)*ω*.

**Figure 3:**
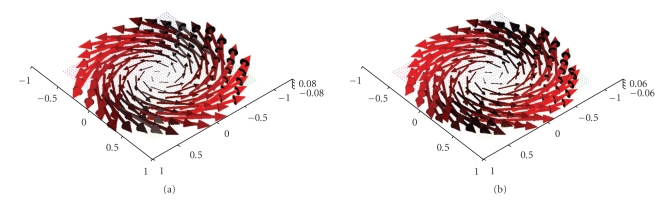
(a) Original vector field **f**(*x*) = (−*x*_2_, *x*_1_, 0)^⊤^. (b) Reconstruction with the described algorithm for *γ* = 0.00692 using exact, simulated data **D**_1_ **f**.

**Figure 4:**
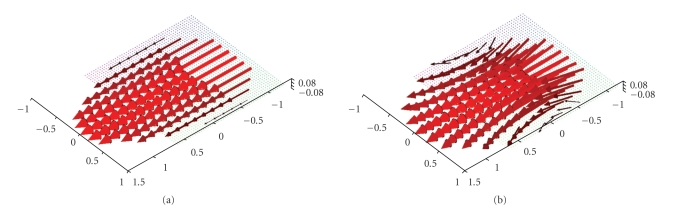
(a) Original vector field **f**(*x*) = (1 − *x*_2_^2^ − *x*_3_^2^, 0, 0)^⊤^. (b) Reconstruction with the described algorithm for *γ* = 0.007 using exact, simulated data **D**_1_ **f**.

**Figure 5:**
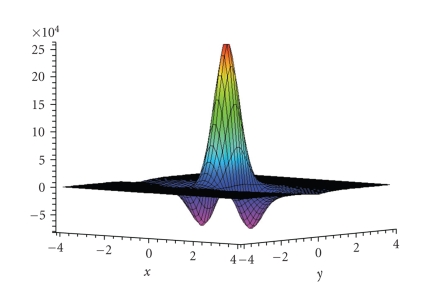
Reconstruction kernel *v*_*γ*_(0; *α*, *ω*) for *γ* = 0.007 associated to the scalar cone beam transform **D**_0_ according to [12].

Figures 3 and 4 display first results of the above algorithm when applied to exact, simulated data for the solenoidal vector fields **f**(*x*) = (−*x*_2_, *x*_1_, 0)^⊤^ and **f**(*x*) = (1 − *x*_2_^2^ − *x*_3_^2^, 0, 0)^⊤^. As already said, we made use of the measurement setup as shown in Figure 1. The scanning curve was Γ = *r* *S* ^2^ ∩ {*x*_3_ = 0}, *r* = 3, that is a circle of radius *r* = 3 in the plane {*x*_3_ = 0}. The divergence-free vector fields are assumed to be defined in the three-dimensional unit ball, that is, according to our definitions we have Ω = Ω^3^. The reconstructions depicted in Figures 3 and 4 were done in the plane {*x*_3_ = 0}. The mollifier *e*_*γ*_ defining the fields **E**_*γ*_^*j*^ was chosen as the Gaussian given in (18). The regularization parameter was *γ* = 0.00692 and *γ* = 0.007, respectively. The corresponding reconstruction kernel *v*_*γ*_(0; ·, ·) is depicted in Figure 5. The reconstruction kernel can be seen as a lowpass filter. Then, the regularization parameter *γ* determines the width of the filter and thus can be interpreted as the cutoff frequency. Large values of *γ* correspond to a large smoothing effect in the reconstructed vector field. Unfortunately we cannot estimate the range of an optimal *γ* since it depends on the noise level of the measured data as well as on the exact solution **f** itself. Nevertheless, in our experiments a value of *γ* ≈ 0.007 always led to good results.

**Figure 6:**
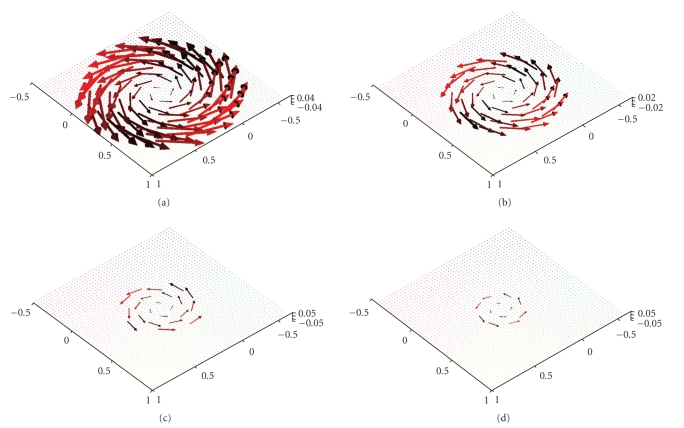
Reconstruction of the vector field **f**(*x*) = (−*x*_2_, *x*_1_, 0)^⊤^ with the described algorithm for *γ* = 0.00692 using exact, simulated data **D**_1_ **f**. (a),(b) The planes {*x*_3_ = 0.5} and {*x*_3_ = 0.75}. (c),(d) The planes {*x*_3_ = 0.9} and {*x*_3_ = 0.95}.

**Figure 7:**
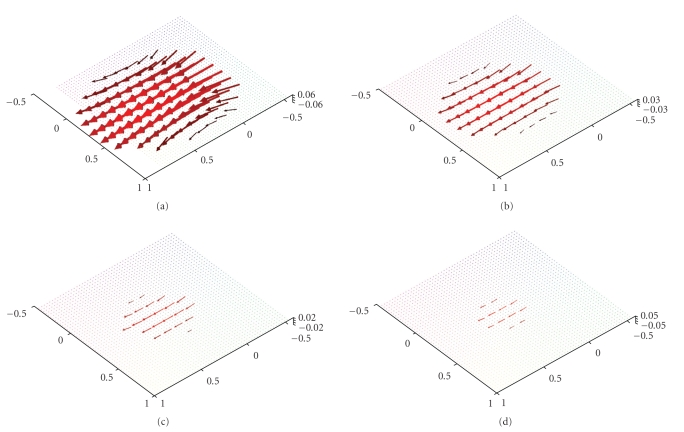
Reconstruction of the vector field **f**(*x*) = (1 − *x*_2_^2^ − *x*_3_^2^, 0, 0)^⊤^ with the described algorithm for *γ* = 0.007 using exact, simulated data **D**_1_ **f**. (a),(b) The planes {*x*_3_ = 0.5} and {*x*_3_ = 0.75}. (c),(d) The planes {*x*_3_ = 0.9} and {*x*_3_ = 0.95}.

Figure 4 emphasizes that the main part of the reconstruction error is located at the boundary of the domain. Although our scanning curve is only one circle our algorithm nevertheless allows us to reconstruct any arbitrary plane in the *x*_3_-direction. Figures 6 and 7 show the reconstructions for the planes {*x*_3_ = 0.5}, {*x*_3_ = 0.75}, {*x*_3_ = 0.9} and {*x*_3_ = 0.95} of the two afore-mentioned vector fields. Figure 7 also illustrates that the intensity of the vector field **f**(*x*) = (1 − *x*_2_^2^ − *x*_3_^2^, 0, 0)^⊤^ decreases as should be expected since *x*_3_^2^ is subtracted in the first component and that even the directional error at the boundary is reduced with increasing *x*_3_.

**Figure 8:**
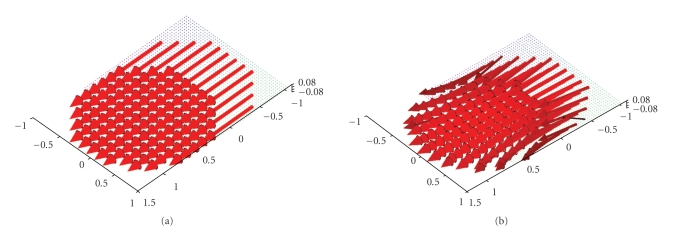
(a) Original vector field **f**(*x*) = (1, 0, 0)^⊤^. (b) Reconstruction with the described algorithm for *γ* = 0.0075 using exact, simulated data **D**_1_ **f**.

**Figure 9:**
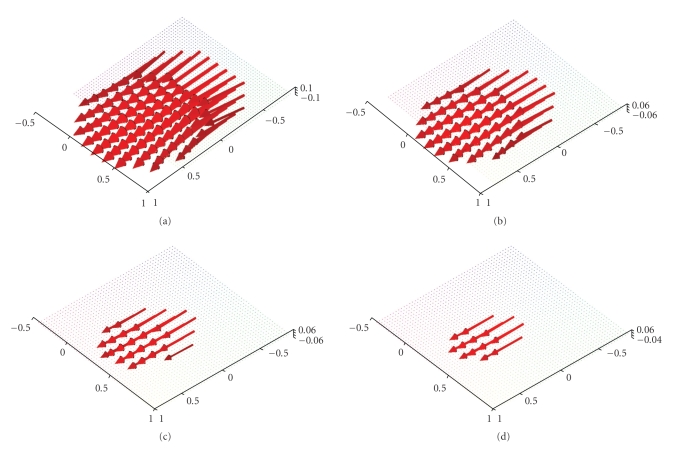
Reconstruction of the vector field **f**(*x*) = (1, 0, 0)^⊤^ with the described algorithm for *γ* = 0.0075 using exact, simulated data **D**_1_ **f**. (a),(b) The planes {*x*_3_ = 0.5} and {*x*_3_ = 0.75}. (c),(d) The planes {*x*_3_ = 0.9} and {*x*_3_ = 0.95}.

The very simple vector field **f**(*x*) = (1, 0, 0)^⊤^ allows us to gain more insight into possible problems and limitations of our algorithm. Figure 8 depicts the original vector field and the reconstruction in the plane {*x*_3_ = 0} just as for the other vector fields before. The regularization parameter was *γ* = 0.0075. There is approximately the same directional error at the boundary as in Figure 4. In addition to that, a slight error in intensity becomes visible. As before, the reconstruction for different planes in the *x*_3_-direction is shown in Figure 9. As in the reconstruction of the field **f**(*x*) = (1 − *x*_2_^2^ − *x*_3_^2^, 0, 0)^⊤^, it is clearly visible that the directional error at the boundary of the field is reduced and that we obtain a uniform direction of the arrows the farther we move away from the plane {*x*_3_ = 0}. But the images also show that the intensity of the vector field is slightly decreasing with increasing *x*_3_ which should certainly not be the case for this particular vector field. In our ongoing studies of the reconstruction algorithm we try to avoid this problem by either using a varying scaling factor for the different planes or by using a different regularization parameter.

**Figure 10:**
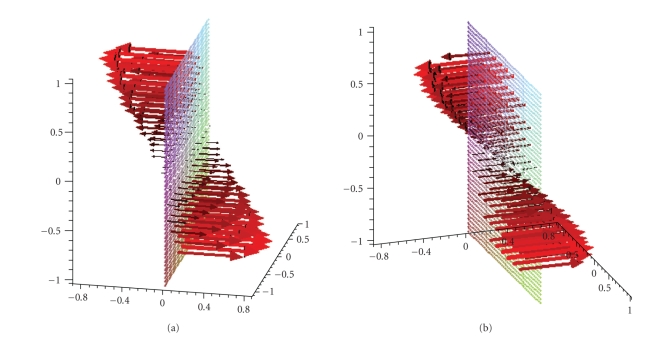
Reconstruction of the vector field **f**(*x*) = (−*x*_2_, *x*_1_, 0)^⊤^ with the described algorithm for *γ* = 0.00692 in the plane {*x*_1_ = 0}.

**Figure 11:**
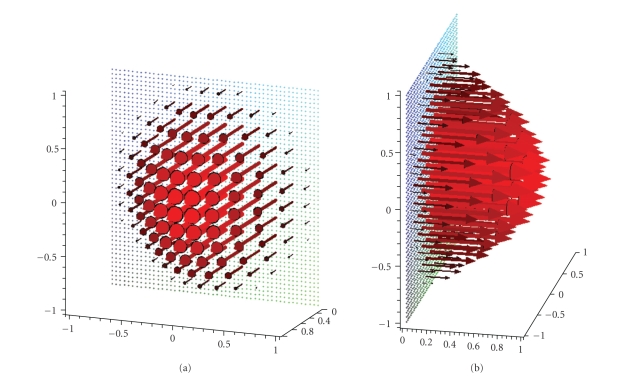
Reconstruction of the vector field **f**(*x*) = (1 − *x*_2_^2^ − *x*_3_^2^, 0, 0)^⊤^ with the described algorithm for *γ* = 0.007 in the plane {*x*_1_ = 0}.

**Figure 12:**
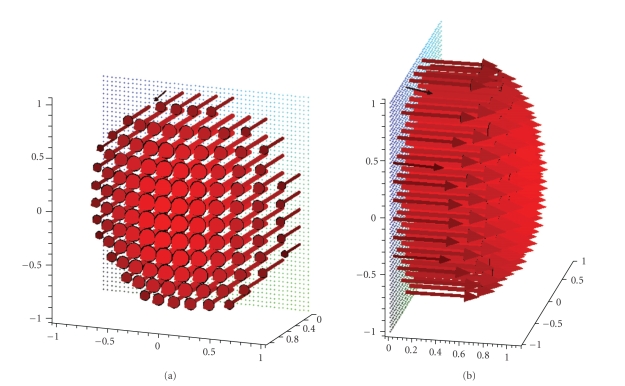
Reconstruction of the vector field **f**(*x*) = (1, 0, 0)^⊤^ with the described algorithm for *γ* = 0.0075 in the plane {*x*_1_ = 0}.

Since the described algorithm allows the reconstruction of any arbitrary plane in the *x*_3_-direction, a vertical cross-section of a vector field can easily be computed. Figure 10 shows a reconstruction of the plane {*x*_1_ = 0} for the circular vector field **f**(*x*) = (−*x*_2_, *x*_1_, 0)^⊤^ for two different viewing angles. Figures 11 and 12 display the analogous results for the vector fields **f**(*x*) = (1 − *x*_2_^2^ − *x*_3_^2^, 0, 0)^⊤^ and **f**(*x*) = (1, 0, 0)^⊤^, respectively. In Figure 11 we recognize a laminar flow just as it would be expected for the flow in blood vessels whereas Figure 12 once more reveals the undesired decrease in intensity.

**Figure 13:**
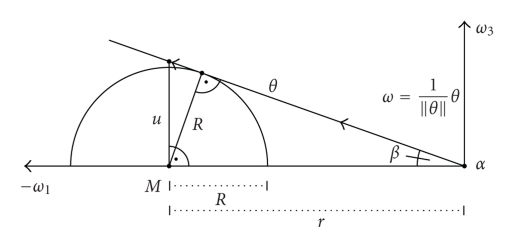
Clarifying the calculation of the maximal value for *ω*_3_.

Up to now, we have only shown reconstructions of divergence-free vector fields which were perpendicular to (0, 0, 1)^⊤^, planar solenoidal fields so to speak. This was done because reconstructing the plane {*x*_3_ = 0} we have to face another difficult problem. Considering the plane {*x*_3_ = 0} it is obvious that the third component of the direction vector *ω* must always be zero, that is, *ω* = (*ω*_1_, *ω*_2_, 0)^⊤^. From formula (3) we can easily calculate the projection of the vector field **f** onto *ω* ∈ {*x*_3_ = 0} as

(32) $$\begin{array}{ll} D_{1}f(\alpha ,\omega ) & \;\;=\int_{0}^{\infty }\langle f(\alpha +t\omega ),\omega \rangle \text{d}t\\ & \;\;=\int_{0}^{\infty }(f_{1}(\alpha +t\omega )\omega_{1}+f_{2}(\alpha +t\omega )\omega_{2}\\ & \;\;\;\;+f_{3}(\alpha +t\omega )\omega_{3})\text{d}t\\ & \overset{\omega_{3}=0}{=}\int_{0}^{\infty }\left(f_{1}(\alpha +t\omega )\omega_{1}+f_{2}(\alpha +t\omega )\omega_{2}\right)\text{d}t, \end{array}$$

which means that the vertical component *f*_3_(*x*) of any vector field cannot be reconstructed in that particular case. But even for planes where *x*_3_ ≠ 0, the reconstruction of the third component of a vector field will be very difficult. This becomes clear if we look at each of the values *ω*_1_, *ω*_2_ and *ω*_3_ of our direction vector *ω* = (*ω*_1_, *ω*_2_, *ω*_3_)^⊤^ separately. Clearly, *ω*_1_ and *ω*_2_ take all values from −1 to 1 if the source travels around the circular scanning curve. This does not apply to *ω*_3_. The maximal value for *ω*_3_ is obtained for the tangential ray with *ω*_2_ = 0, that is the maximal ray in the *x*_1_-*x*_3_-plane hitting the object Ω in just one point. From Figure 13 we easily see that

(33) $$\begin{array}{ll} \sin\;(\beta ) & =\frac{R}{r},\\ \tan\;(\beta ) & =\frac{u}{r}⟺u=r\;\tan\;(\beta )=r\;\tan\;\left(\arcsin\;\left(\frac{R}{r}\right)\right). \end{array}$$

Using the equivalence

(34) $$\arcsin\;(x)=\arctan\;\left(\frac{x}{\sqrt{1-x^{2}}}\right)$$

we obtain

(35) $$\begin{array}{ll} u & =r\tan\;\left(\arctan\;\left(\frac{R/r}{\sqrt{1-{\left(R/r\right)}^{2}}}\right)\right)\\ & =\frac{R}{\sqrt{(r^{2}-R^{2})/r^{2}}}=\frac{rR}{\sqrt{r^{2}-R^{2}}}. \end{array}$$

For the direction vector *ω* we can then deduce

(36) $$\begin{array}{cc} \omega & =\frac{1}{∥\theta ∥}\theta \;\text{with}\;\;\theta =\left(\begin{array}{c} -r\\ 0\\ u \end{array}\right),\\ ∥\theta ∥ & =\sqrt{r^{2}+u^{2}}=\sqrt{r^{2}+\frac{{\left(rR\right)}^{2}}{r^{2}-R^{2}}}=r\sqrt{1+\frac{R^{2}}{r^{2}-R^{2}}}\\ & =r\sqrt{\frac{r^{2}-R^{2}+R^{2}}{r^{2}-R^{2}}}=\frac{r^{2}}{\sqrt{r^{2}-R^{2}}},\\ \frac{u}{\sqrt{r^{2}+u^{2}}} & =\frac{\left(rR/\sqrt{r^{2}-R^{2}}\right)}{\left(r^{2}/\sqrt{r^{2}-R^{2}}\right)}=\frac{R}{r},\\ \frac{-r}{\sqrt{r^{2}+u^{2}}} & =\frac{-r}{\left(r^{2}/\sqrt{r^{2}-R^{2}}\right)}=-\frac{\left(\sqrt{r^{2}-R^{2}}\right)}{r}\\ \Rightarrow \omega & =\left(\begin{array}{c} -\frac{\left(\sqrt{r^{2}-R^{2}}\right)}{r}\\ 0\\ \frac{\begin{array}{c} R \end{array}}{r} \end{array}\right). \end{array}$$

This simple geometric calculation shows that the angle *β* between the plane {*x*_3_ = 0} and the tangential ray is not exceeding ≈ 19.47° for our geometric setup in which the object is contained in the three-dimensional unit ball Ω^3^, so *R* = 1, and the scanning circle Γ has radius *r* = 3. We conclude that

(37) $$-\frac{R}{r}\leq \omega_{3}\leq \frac{R}{r}⟺-\frac{1}{3}\leq \omega_{3}\leq \frac{1}{3}.$$

The problem with the small values for *ω*_3_ gets even more difficult the larger the distance between object and scanning circle is chosen. This fact might even be responsible for the intensity error observed when reconstructing different planes in the *x*_3_-direction as mentioned above.

**Figure 14:**
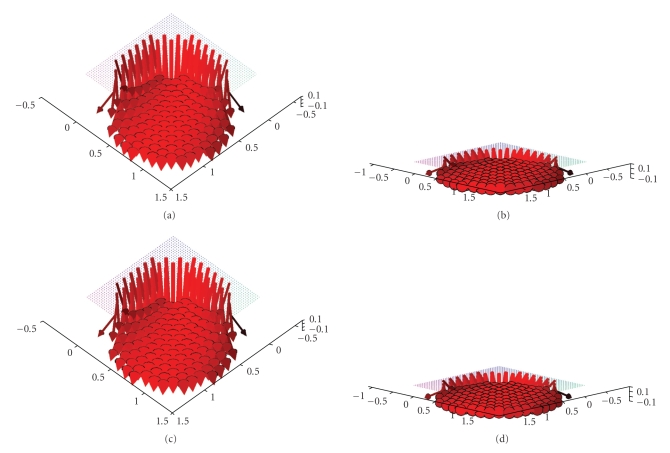
(a),(b) Reconstruction of the vector field **f**(*x*) = (1, 1, 1)^⊤^ with the described algorithm for *γ* = 0.007 in the plane {*x*_3_ = 0}. (c),(d) Reconstruction of the vector field **f**(*x*) = (1, 1, −1)^⊤^ with the described algorithm for *γ* = 0.007 in the plane {*x*_3_ = 0}.

**Figure 15:**
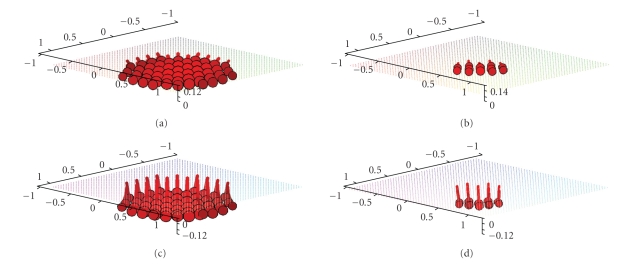
Top: reconstruction of the vector field *f*(*x*) = (1, 1, 1)^⊤^ with the described algorithm for *γ* = 0.007 in the planes (a) {*x*_3_ = 0.75} and (b) {*x*_3_ = 0.95}. Bottom: reconstruction of the vector field *f*(*x*) = (1, 1, −1)^⊤^ with the described algorithm for *γ* = 0.007 in the planes (c) {*x*_3_ = 0.75} and (d) {*x*_3_ = 0.95}.

The consequences of the problem can easily be illustrated by applying our algorithm to the two fully three-dimensional vector fields **f**(*x*) = (1, 1, 1)^⊤^ and **f**(*x*) = (1, 1, −1)^⊤^ whose *x*_3_-component should obviously point to opposite directions. Figure 14 shows that even for different perspectives there is no visible difference between the two reconstructions in the plane {*x*_3_ = 0}. Only for larger (or smaller) values of *x*_3_, that means planes above (or below) the plane of the scanning curve {*x*_3_ = 0}, the reconstructions become distinguishable. This is shown in Figure 15 where the results for {*x*_3_ = 0.75} and {*x*_3_ = 0.95} can be directly compared to each other. Even in this case the difference is marginal. For the vector field **f**(*x*) = (1, 1, 1)^⊤^ the arrows characterizing the vectors are slightly pointing upwards whereas those of **f**(*x*) = (1, 1, −1)^⊤^ are pointing downwards. This can be seen from the scale as well as from the colored points indicating the reconstruction plane. For the latter vector field these points are painted above the arrows proving that the reconstructed vectors point in a negative *x*_3_-direction.

**Figure 16:**
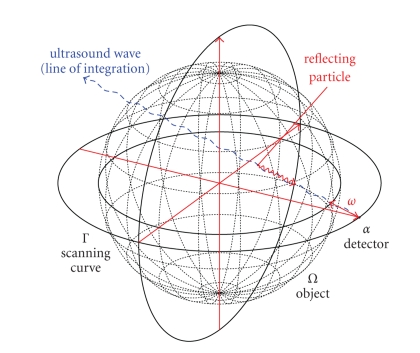
Measurement setup using two orthogonal circles as scanning curve Γ, one in the plane {*x*_3_ = 0} as usual, and one in the plane {*x*_1_ = 0}.

**Figure 17:**
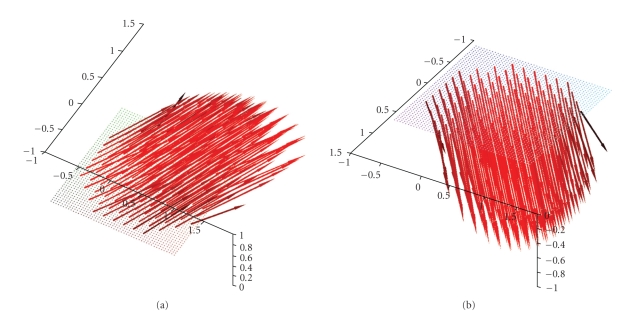
(a) Reconstruction of the vector field **f**(*x*) = (1, 1, 1)^⊤^ with the described algorithm for *γ* = 0.007 with circles in the planes {*x*_3_ = 0} and {*x*_1_ = 0}. (b) Reconstruction of the vector field **f**(*x*) = (1, 1, −1)^⊤^ with the described algorithm for *γ* = 0.007 with circles in the planes {*x*_3_ = 0} and {*x*_1_ = 0}.

Despite all these drawbacks it is nevertheless possible to reconstruct a whole three-dimensional vector field. Summarizing the results so far, the *x*_1_- and *x*_2_-component of any vector field **f**(*x*) = (*f*_1_, *f*_2_, *f*_3_)^⊤^, that is, *f*_1_ and *f*_2_, can be reconstructed for any plane in the *x*_3_-direction. Changing our measurement setup by adding to the current scanning curve Γ = *r* *S* ^2^ ∩ {*x*_3_ = 0}, *r* = 3, a second, orthogonal circle of the same radius in the plane {*x*_1_ = 0} enables us to reconstruct the *x*_2_- and *x*_3_-component of any vector field, that is *f*_2_ and *f*_3_, by using our algorithm as usual. Lade et al. used a comparable setup of two “perpendicular tilt series” in [21] for their longitudinal and transverse measurements. For this additional circle we compute the vertical cross-section of the field in the appropriate plane as shown before. The computations can be done simultaneously which means that no additional time is needed. The modified measurement setup is depicted in Figure 16. As we have shown, we are able to reconstruct *f*_1_ and *f*_2_ for any plane in the *x*_3_-direction with the algorithm presented at the beginning of the paper. The second, orthogonal circle in the plane {*x*_1_ = 0} enables us to analogously reconstruct *f*_2_ and *f*_3_ for any plane in the *x*_1_-direction. Taking a vertical cross-section at *x*_3_ = 0, both results can be combined to reconstruct a complete slice of a three-dimensional vector field. Thereby, we have to pay attention to compute the arithmetic mean for the *f*_2_-component since it is correctly reconstructed for both measurements. First numerical results are depicted in Figure 17, where the vector fields **f**(*x*) = (1, 1, 1)^⊤^ and **f**(*x*) = (1, 1, −1)^⊤^ have been reconstructed by means of the described method. In contrast to the previous images the width of the arrows has been changed to improve the recognizability of the various details.

**Figure 18:**
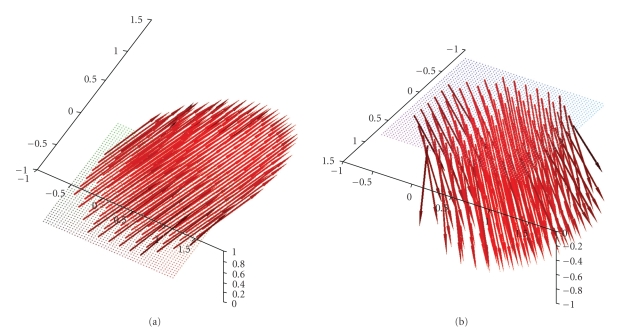
(a) Reconstruction of the vector field **f**(*x*) = (1, 1, 1)^⊤^ with the described algorithm for *γ* = 0.007 with circles in the planes {*x*_3_ = 0} and {*x*_2_ = 0}. (b) Reconstruction of the vector field **f**(*x*) = (1, 1, −1)^⊤^ with the described algorithm for *γ* = 0.007 with circles in the planes {*x*_3_ = 0} and {*x*_2_ = 0}.

It should be added that it is also possible to choose the second orthogonal circle to lie in the plane {*x*_2_ = 0} instead of {*x*_1_ = 0}. This leads to some minor changes in the appearance of the reconstructed images which can be seen from Figure 18. It seems as if each of the vertical circles has its advantages in the reconstruction of a certain direction and as such one or the other may be better suited if some prior knowledge of the vector field exists.

**Figure 19:**
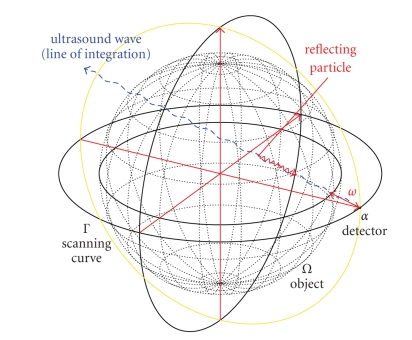
Measurement setup using all three orthogonal circles as scanning curve Γ, one in each plane {*x*_*i*_ = 0}, *i* = 1, 2, 3.

**Figure 20:**
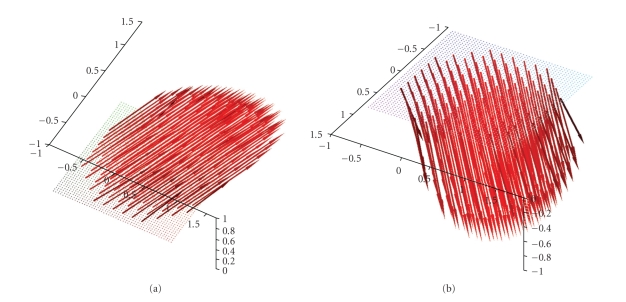
(a) Reconstruction of the vector field **f**(*x*) = (1, 1, 1)^⊤^ with the described algorithm for *γ* = 0.007 using three orthogonal circles in the planes {*x*_*i*_ = 0}, *i* = 1, 2, 3. (b) Reconstruction of the vector field **f**(*x*) = (1, 1, −1)^⊤^ with the described algorithm for *γ* = 0.007 using three orthogonal circles in the planes {*x*_*i*_ = 0}, *i* = 1, 2, 3.

Moreover, we can combine the advantages of both scanning geometries suited for fully three-dimensional reconstruction we introduced so far. This can be done by simply using data from all three orthogonal circles at once. This obviously leads to a certain redundancy in the data which might nevertheless be useful to improve the quality of our reconstructions. The yellow circle in Figure 19 is not necessary to obtain a complete 3D reconstruction of the vector field, it is only meant to supply us with additional information. Reconstructions of the two vector fields **f**(*x*) = (1, 1, 1)^⊤^ and **f**(*x*) = (1, 1, −1)^⊤^ using the three orthogonal circles can be found in Figure 20. Comparing them with the images made by using only two circles as scanning curve we can see that especially the direction but also the length of the arrows is reconstructed much better.

**Figure 21:**
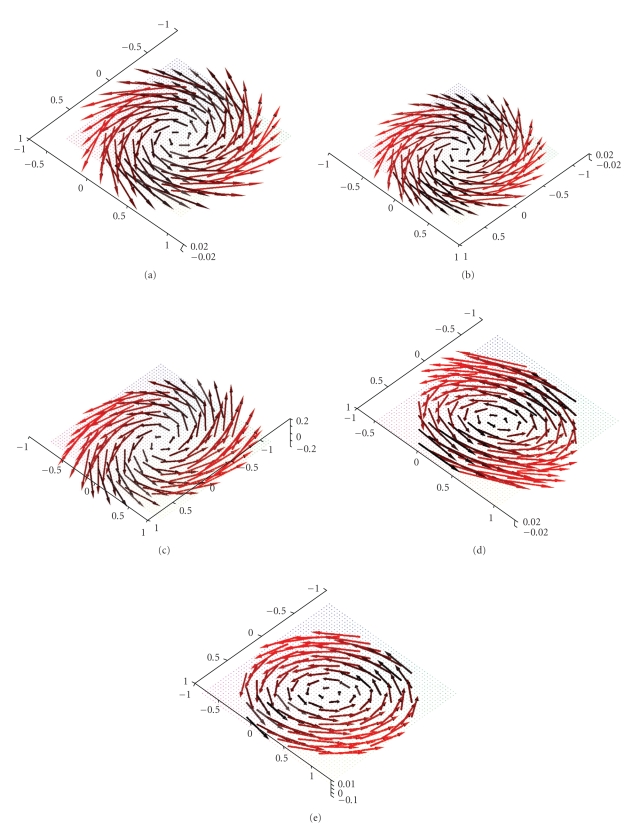
(a) Original vector field **f**(*x*) = (−*x*_2_, *x*_1_, 0)^⊤^. (b) Reconstruction with the described algorithm for *γ* = 0.00692 using one single circle in the plane {*x*_3_ = 0}. (c) Reconstruction with the described algorithm for *γ* = 0.00692 using circles in the planes {*x*_3_ = 0} and {*x*_1_ = 0}. (d) Reconstruction with the described algorithm for *γ* = 0.00692 using circles in the planes {*x*_3_ = 0} and {*x*_2_ = 0}. (e) Reconstruction with the described algorithm for *γ* = 0.00692 using three orthogonal circles in the planes {*x*_*i*_ = 0}, *i* = 1, 2, 3.

To verify our assumptions we reconstructed once again the planar circular vector field **f**(*x*) = (−*x*_2_, *x*_1_, 0)^⊤^. Comparing the images in Figure 21, where the width of the arrows was reduced in comparison to the ones from the beginning of the paper, we see that using all three orthogonal circles is by far better than using only two of them. Unfortunately we have to admit that the three-dimensional reconstruction is not as good as the one obtained by only one circle in the plane {*x*_3_ = 0}. This can be explained by the fact that the regularization parameter *γ* was optimized for the latter scanning geometry and was then used for all further modifications of the measurement setup as well. Thus, further enhancements of the results are to be expected by choosing a varying regularization parameter for the different circular scanning geometries. In addition to that we might implement a scaling factor to compensate for the loss of intensity at the boundary of our reconstruction region. It might also be possible to incorporate some sort of prior knowledge. For example in clinical applications when measuring blood flow we may use some information about the blood vessels to correct the direction of the flow at the boundary.

It is to mention that in contrast to our initial scanning geometry of only one circle the two as well as the three orthogonal circles certainly meet the requirements of Tuy-Kirillov's condition [27, 28], namely that the source curve Γ intersects each plane hitting supp(*f*) transversally, see [17]. This might be useful for future theoretical advances in the field of three-dimensional vector tomography as well as for improvements and extensions to our algorithm. This is part of our current research.

## 4. CONCLUSION

We presented a first approach for reconstructing cone beam data in three-dimensional vector field tomography. The algorithm relies on known results for the scalar case from [12, 26], where the method of approximate inverse has been applied for the computation of reconstruction kernels for circular 3D cone beam tomography. A possibility to extend that very efficient regularization technique to VFT has been shown and first numerical experiments are very promising.

The investigation of what happens when we use a scanning curve Γ different from what has been presented in this paper is subject of current research. Nevertheless, it is to mention that the proposed algorithm is not restricted to circles as scanning curves. Helical scanning geometries are especially interesting in clinical applications because a helical source trajectory can easily be implemented by moving the patient's bed through the scanner's gantry at constant speed, a method which is common practice in cone beam CT scanning today. Although data acquisition itself is more difficult, it is much faster and thus better suited for clinical diagnostics. The results of Katsevich [29] could help when we consider a helix as trajectory. Reconsidering the generalizations to tensor fields of arbitrary rank and in arbitrary dimension from which we refrained in the first section of the paper, it is to mention that Denisjuk [30] formulated a generalization of Tuy-Kirillov's condition to tensor fields of rank *m* which might help to compute exact reconstruction kernels for **D**_*m*_. Denisjuk also proved in [30] that a full reconstruction of solenoidal vector fields is possible if the vectorial cone beam data **D**_1_ **f**(*α*, *ω*) are available for all *α* on a trajectory Γ satisfying a generalized Tuy condition and all directions *ω* ∈ *S* ^2^.

Furthermore, the problem of signal attenuation which is important for Doppler tomography using ultrasound has been addressed by several authors. Unfortunately, so far only works have been published dealing with the problem in two dimensions. Bukhgeim and Kazantsev derived a 2D inversion formula in [31]. Their proof uses a coordinate transformation into complex variables which cannot be generalized to three dimensions. In [32], Natterer develops an extension of Novikov's inversion formula for 2D vector fields. Finally, Stråhlén proves a Fourier slice theorem for the attenuated vectorial Radon transform in two dimensions for the parallel geometry in [33]. Further advances in this field of research will certainly be interesting for 3D cone beam vector field tomography as well.

By now the data acquisition in vector field tomography neglects the wave structure of the ultrasound signals. Further research might take the wave structure into account to improve the reconstruction and modeling accuracy. In this respect the application of the Rayleigh-Sommerfeld formula could be useful.

## Figures and Tables

**Algorithm 1:**
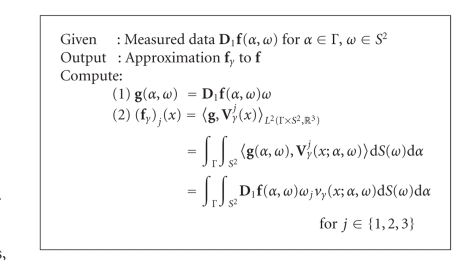
For cone beam VFT.
